# Unusual explosive growth of a squamous cell carcinoma of the scalp after electrical burn injury and subsequent coverage by sequential free flap vascular connection – a case report

**DOI:** 10.1186/1471-2407-5-150

**Published:** 2005-11-28

**Authors:** Raymund E Horch, G Bjoern Stark, Justus P Beier

**Affiliations:** 1 Department of Plastic and Hand Surgery, University Hospital of Erlangen-Nuernberg, Friedrich-Alexander-University Erlangen-Nuernberg, Germany; 2 Department of Plastic and Hand Surgery, University Hospital of Freiburg, Albert-Ludwigs-University Freiburg, Germany

## Abstract

**Background:**

Squamous cell carcinomos may arise from chronic ulcerating wounds in scars, most commonly postburn scars. Tumour growth usually takes place over months to years. Localization on the scalp is a relatively rare condition.

**Case presentation:**

This report presents the case of a 63-year-old man with chronic ulceration of a postburn scar of the scalp due to an electrical burn 58 years ago. Sudden tumour growth started within weeks and on presentation already had extended through the skull into frontal cortex. After radical tumour resection, defect was covered with a free radial forearm flap. Local recurrence occurred 6 weeks later. Subsequent wide excision including discard of the flap and preservation of the radial vessels was followed by transfer of a free latissimus dorsi muscle flap, using the radial vessels of the first flap as recipient vessels. The patient received radiotherapy post-operatively. There were no problems with flap survivals or wound healing. Due to rapidly growing recurrence the patient died 2 months later.

**Conclusion:**

Explosive SCC tumour growth might occur in post-burn scars after more than 50 years. As a treatment option the use of sequential free flap connections might serve in repeated extensive tumour resections, especially in the scalp region, where suitable donor vessels are often located in distance to the defect.

## Background

Malignant degeneration of burn scars has been reported as early as in the first century by Celsus. However, the classic description was published by Jean-Nicholas Marjolin in 1828, who indeed did not regard the lesion as malignant [1]. The entity „Marjolin's ulcer“, which is commonly used for carcinoma arising in post-traumatic scars, has been applied ever since the report of Da Costa in 1903, who first used the term [2]. Today the term "Marjolin's ulcer" is generally used for squamous cell carcinoma arising in posttraumatic scars. However squamous cell carcinoma has recently been reported to arise also from other skin lesions such as Fournier's gangrene[3]. There have been some case reports of burn scar carcinomas of the scalp [4-6], a localization not as common as the lower extremity but still making up approximately 14% of burn scar carcinomas[7]. Due to involvement of the skull and brain, wide excision can require complex reconstructions including free flap transfer. Utilizing previously anastomosed recipient vessels of an earlier transposed free flap as new donor vessels, may serve as an ideal tool for secondary free flap surgery in cases of local recurrence, as in the reported case.

## Case presentation

**Figure 1:**
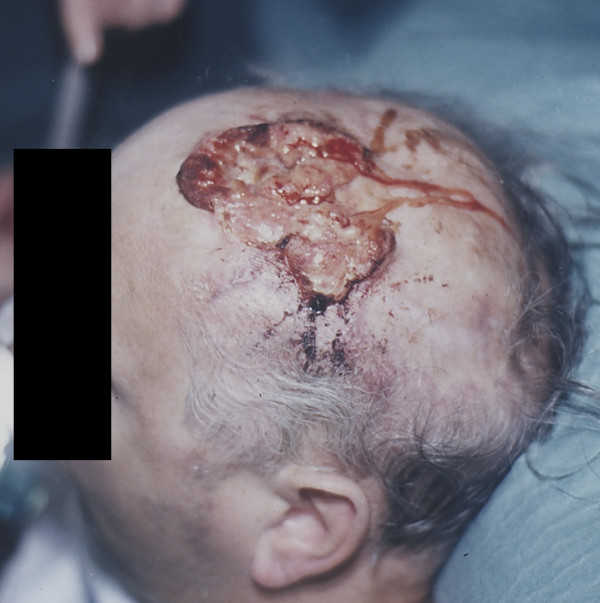
63 year old male patient presenting with a large 8 × 8 cm measuring exulcerated suppurating tumor of the scalp (Marjolin's ulcer).

A 63 year old male patient presented with a large 8 × 8 cm ulcerated and suppurating tumour of the scalp (Fig. 1). The patient had suffered from an electrical burn at the age of 5 with delayed and incomplete healing of the wound and subsequent baldness in the healed area. The wound had never been completely healed and ulcerated again 8 weeks before admission to the hospital. The patient, a professor of philosophy, wore a turban for decades to hide the chronic wound. Due to personal neglect and the circumstance that he had been living on his own for many years, without any relatives or a partner kept him from seeing a doctor. The disease only received medical attention when he suddenly experienced a complete aphasia during lectures.

**Figure 2:**
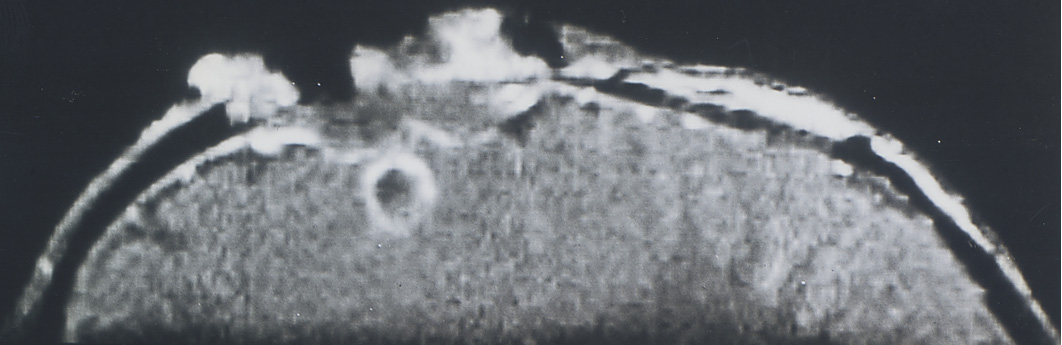
Nuclear magnetic resonance imaging revealing tumor infiltration of the cranium and frontal cortex.

**Figure 3:**
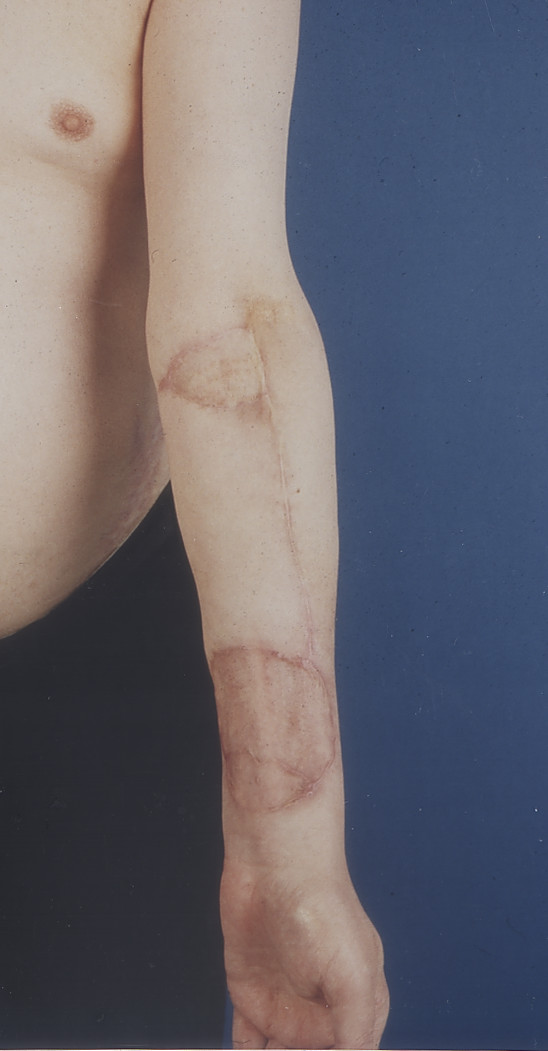
Four weeks postoperative aspect of donor site at left forearm.

**Figure 4:**
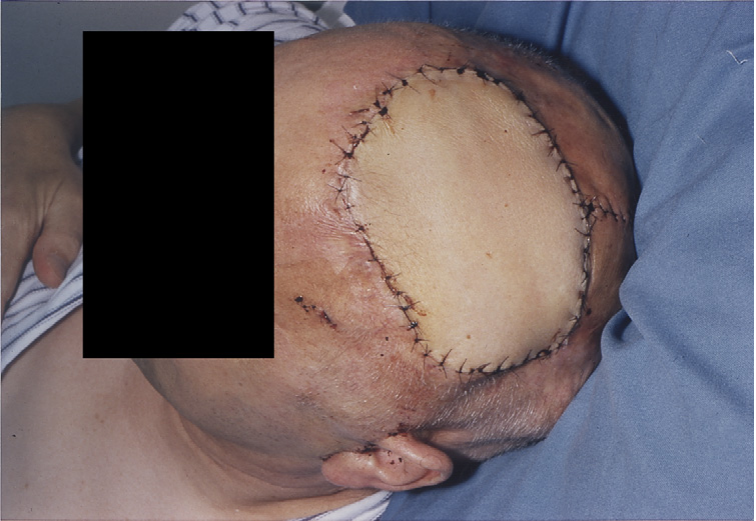
Uncomplicated healing of radial forearm flap on left hemicranium.

**Figure 5:**
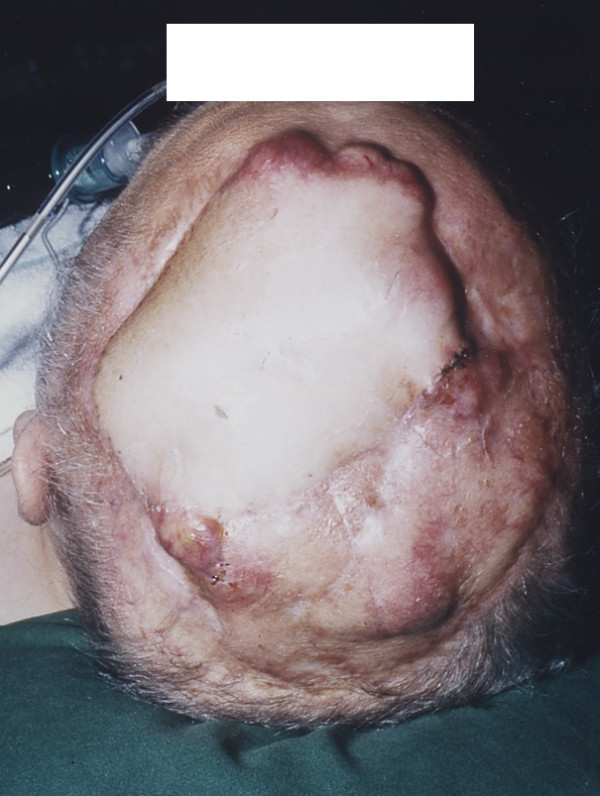
Six weeks after primary resection tumor relapse with multiple satellite metastases around the radial forearm flap.

**Figure 6:**
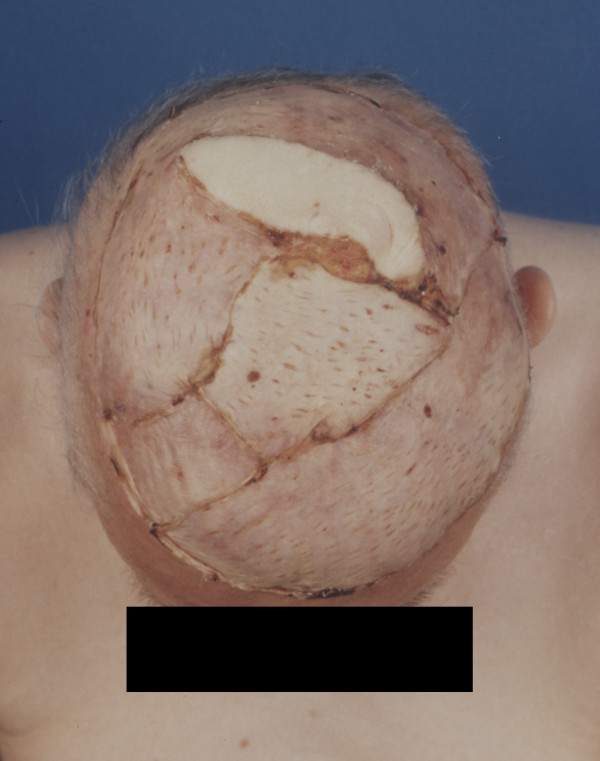
Secondary wide resection and closure of defect with a skin grafted latissimus dorsi myocutaneous flap consecutively connected to the former radial artery and vein of the primary flap.

**Figure 7:**
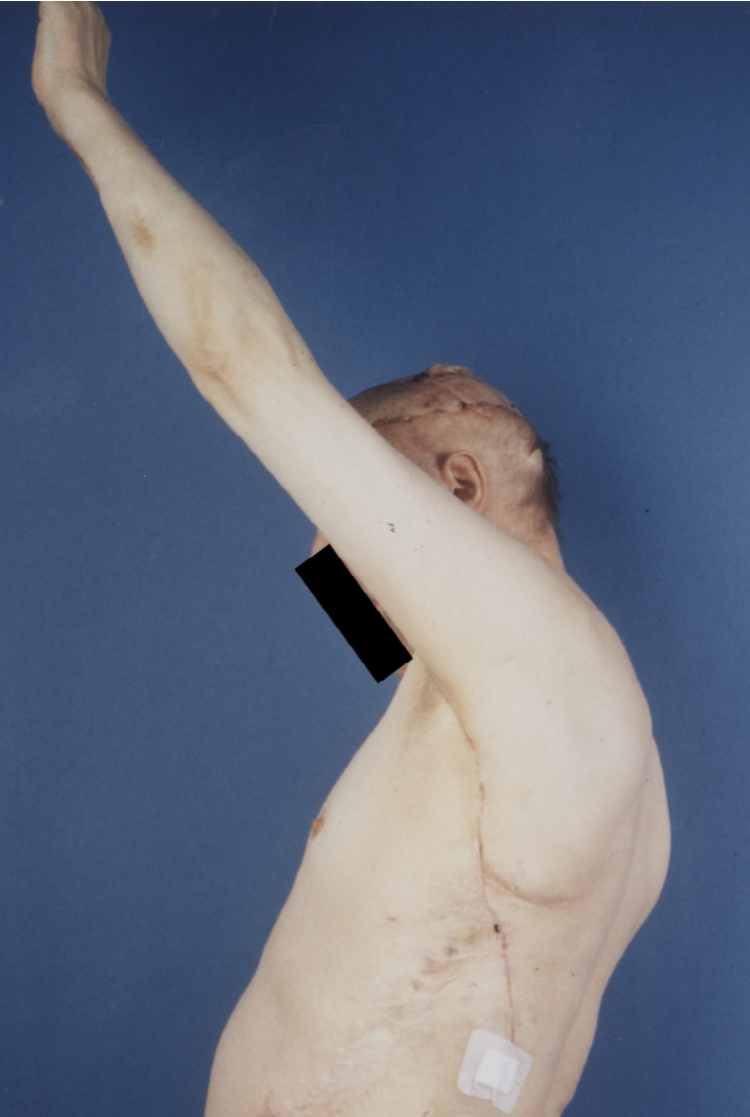
Uneventful early postoperative course after second free flap with uncomplicated donor site and no impairment of shoulder function.

Clinically there was a palpable mass adherent to the skull. Nuclear magnetic resonance and computed tomography imaging revealed a tumour with infiltration of the cranium and the brain (Fig. 2). Radical resection of the tumour including the cranium, as well as dura mater and the affected parts of the frontal cortex was performed. The dural defect was closed with an autologous fascia lata patch from the right thigh, and as recipient vessels, the superficial temporal artery and vein were dissected. A radial forearm flap with a 10 × 10 cm skin paddle was harvested with an adequately long vascular pedicle. Radial artery and vein were anastomosed end-to-end to the superficial temporal vessels above the zygomatic arch. The donor site was covered with a split thickness skin graft from the thigh (Fig. 3). Histology of the specimen revealed a squamous cell carcinoma and free resectional margins. Postoperative healing was uneventful and the patient regained his speech immediately after the operation (Fig. 4). Six weeks later, the patient was referred to our unit again with multiple satellite nodules around the radial forearm free flap (Fig. 5). Incision biopsy revealed recurrence of squamous cell carcinoma. In a second operation, radical excision of the scalp with wide margins was performed, requiring sufficient soft tissue coverage. The skin and soft tissue of the radial forearm flap was completely resected, but the proximal part of the radial vessels which were in sufficient distance from the tumour were preserved. Thus radial artery and vein served as recipient vessels for a latissimus dorsi free flap. After the second operation postoperative healing was uneventful again, and the patient recovered well initially for the first three weeks (Fig. 6 and 7). Postoperatively, radiation therapy with single focus dose of 14 Gray was administered and one cycle of combined chemotherapy was conducted with cisplatin 180 mg i.v. on day 1 and 5-fluorouracil 1800 mg i.v. on days 1–5 continuously. However despite these efforts, six weeks after the second resection the patient again developed multiple recurrent metastatic lesions around the latissimus dorsi flap, and cerebral symptoms. Clinically, the general condition of the patient dramatically worsened and he died within two more weeks.

## Conclusion

In this case report we describe a surgical approach to a recurrent squamous cell carcinoma of the scalp that resulted in particular problems for recontructive surgery. Our approach involved the sequential transfer of a free radial forearm flap after first tumour resection and the subsequent use of the radial vessels as recipient vessels for a free latissimus dorsi flap after second tumour resection. In general treatment of squamous cell carcinoma developing in scars and chronic wounds includes wide local excision, which in many cases demands either extensive soft tissue coverage or even amputation if extremities are involved. Burn scar carcinomas of the scalp are relatively rare compared to extremity localization and primary intracranial malignancies have been falsely mistaken for those lesions [8].

When there is clinical involvement, lymph node dissection is necessary [9]. Marjolin's ulcers have been described as aggressive and are associated with high rates of nodal metastases and a poor prognosis. Two clinical types with different growth patterns are distinguished: on one hand the flat, indurated, infiltrative, ulcerative carcinoma, and on the other hand the exophytic papillary form, which is infrequent and generally less severe. It has also been advocated to subdivide the "Marjolin's ulcers" into 2 variants: acute with a latency of > one year and chronic with an average latency of 36 years[10]. A current meta-analysis determined an average interval between injury and development of Marjolin's ulcer of 32 years (+/- 19 years) [7]. The interval between the initial trauma and the onset of malignacy in our patient confirms this disease pattern of type one, variant two Marjolin's ulcer.

If an old burn scar begins to break down and ulcerate, it has been advocated to consider prophylactic wide local excision and simple skin grafting of the area [5]. For a long time Marjolin's ulcer had not been reported to arise from the site of a skin graft. However, burn scar carcinoma in a previously skin grafted area was described [11]. Deep second degree and in particular third degree burns should be grafted immediately, which can be addressed by different means like split and/or full thickness skin graft transplantation or in very extensive lesions by keratinocyte transplantation [12,13]. Thus lower contracture rates and more sustainable wound healing can be achieved. Burns with unsuccessful grafting procedures are more susceptible to scar cancer formation than burns which have been fully successfully grafted. In the reported case the patient had not received skin grafting during acute phase of the trauma, neither was he treated for chronic wound healing impairment by skin grafting during the following years. Especially in cases of electrical burns, like in the presented case, early estimation and appropriate treatment would have been crucial [14]. Skin grafts would also not have been an option at the stage the patient presented to our clinic. Due to depth of infiltration, resulting in a post-excisional defect that needed durable and immediate soft tissue coverage to seal the subdural space and protect underlying structures, transfer of vascularized soft tissue was mandatory. This concept is similar to other indications in problem wounds as previously reported [15,16].

In general, both size and depth have been recognized to be important factors conjuring up the need for free flap transplantations [17]. In our patient the free radial forearm flap was chosen because of the length of its vascular pedicle and the desirable flatness of a fasciocutaneous flap. Suitability of this flap for the given defect was advocated by other authors [18,19].

After the rapid development of the relapsing tumor we had to create an even larger defect after to achieve complete tumour resection. Thus we were facing the demand for a larger soft tissue cover than after the first tumour resection. The favoring of the free latissimus dorsi muscle flap and its coverage with split thickness skin grafts was based on three advantages of this muscle flap: first all muscle flaps may solve defect problems even under extremely difficult local conditions by their vascularization. Secondly the large diameter of the vessels of the latissimus dorsi flap and its long, anatomically constant vascular pedicle promote safety of this operative procedure. Thirdly, the dimensions and thickness of this large, but flat free muscle flap can be nicely adapted to the convex surface of the skull. Covering the surface of the muscle flap with split thickness skin grafts leads to resistance against chronic mechanical stress and has a better aesthetic result. The particular problem in the presented case was to obtain a sufficient pedicle length, since the contralateral temporal artery was not available for anastomosis after the first tumour resection. Therefore, besides using the latissimus dorsi free flap with its long pedicle, it had to be combined with the preservation and use of the former radial flap vessels as new donor vessels. The concept of utilizing the vascular pedicle of a previous free flap while removing the rest of the flap is an interesting alternative to gain length and freedom of positioning a second required microvascular free flap. One disadvantage in the case of neoplasms involving the first flap might be an increased risk of local recurrence when preserving parts of the first pedicle. In the presented case, we tried to avoid this possibility by only preserving the proximal part of the pedicle.

Only few case reports have described one-step reconstruction with different free flaps sequentially connected in mandibular reconstruction: a fibular osseous free flap was combined with either a radial forearm flap or a lateral arm flap in six patients, with the latter flaps being connected to the distal peroneal vessels [20]. This approach demonstrated the versatility of sequential free flap coverage in cases where vascular access is limited, such as following head and neck surgery and radiation [20]. Sequential free flap transfer has twice been described as an approach to hand reconstruction [21,22]. Serial multiple flap transfers as an operative sequence have also been described as an option in extensive burn reconstruction. In this case series, 2–3 flaps were preferably used from one donor site and transferred to one or two (extensive) defects during one operation [23]. However, these authors did not have to discard the first free flap and he flaps had not been connected consecutively but to two independent donor vessels.

In this case we demonstrate the feasibility of serial microvascular transplantations with removal of the primary flap while preserving the vessels to connect a secondary free flap in complex reconstructions of the scalp. The radial artery and concommitant vein were available as relatively large vessels, matching the thoracodorsal vessels. This approach might therefore be taken into account when planning complex microsurgical reconstructions, especially when discard of the first flap is mandatory like in the presented case.

## Competing interests

The author(s) declare that they have no competing interests.

## Authors' contributions

REH, the principal author was the main operating surgeon in charge of the case manuscript. GBS was the first surgical assistant and the consultant in charge of the case. JPB was the second surgical assistant. He contributed towards preparation of the manuscript.

## Pre-publication history

The pre-publication history for this paper can be accessed here:
